# Regional Differences in the Glycosaminoglycan Role in Porcine Scleral Hydration and Mechanical Behavior

**DOI:** 10.1167/iovs.62.3.28

**Published:** 2021-03-22

**Authors:** Mohammad Pachenari, Hamed Hatami-Marbini

**Affiliations:** 1 Computational Biomechanics Research Laboratory, Mechanical and Industrial Engineering Department, University of Illinois at Chicago, Chicago, Illinois, United States

**Keywords:** glycosaminoglycan, hydration, mechanical properties, porcine sclera

## Abstract

**Purpose:**

This study characterized the role of glycosaminoglycans (GAGs) in the hydration, thickness, and biomechanical properties of posterior and anterior porcine sclera.

**Methods:**

The scleral discs and strips were obtained from the anterior and posterior parts of porcine eyes, and their initial hydration and thickness were measured. The anterior and posterior scleral discs were used to show the efficacy of the GAG removal protocol by quantifying their GAG content. The strips were divided into three groups of PBS treatment, buffer treatment, and enzyme treatment in order to assess the effects of different treatment procedures on the thickness, hydration, and viscoelastic properties of the samples. The mechanical properties of the strips were determined by performing uniaxial tensile stress relaxation experiments.

**Results:**

It was found that the control and buffer groups had insignificant differences in all measured quantities. The samples from the posterior region had a significantly larger GAG content and thickness in comparison with those from anterior region; however, there was an insignificant difference in their hydration. The GAG depletion process decreased the hydration of both anterior and posterior samples significantly (*P* < 0.05). Furthermore, the mechanical tests showed that the removal of GAGs resulted in stiffer mechanical behavior in both anterior and posterior samples (*P* < 0.05). In particular, the peak stress and equilibrium stress were significantly larger for the strips in the enzyme treatment group.

**Conclusions:**

GAGs and their interaction with the collagen network are important in defining the hydration and mechanical properties of both posterior and anterior sclera.

The sclera has a resilient and complex structure that performs several functions essential for the integrity of the optical components of the eye.^1^^,^^2^ The specific microstructure of the scleral extracellular matrix (ECM), such as its large-diameter collagen fibers, creates an opaque fibrous tissue that could prevent internal light scattering within the eye globe.^3^ Sclera also provides a firm substrate without substantial distortion when the eyeball is rotated by the extraocular muscles.^4^ Furthermore, the right scleral curvature is necessary for proper function of the visual system. It is expected that a change in scleral viscoelasticity would have a great impact on visual impairment.^2^ Alterations in the scleral biomechanical properties have been associated with the onset and further progression of pathological conditions and disorders such as myopia and glaucoma.^5^^–^^7^ Myopia is a negative refractive error, produced by an excessive lengthening of the posterior segment, which pulls the retina behind the focal length of the eye.^8^^,^^9^ In addition to myopia, high intraocular pressure is the most critical factor in glaucoma and results in increased deformation in the optic nerve head (ONH), which is the early site of glaucomatous damage.^10^ Many investigations have observed that scleral mechanical properties have a significant effect on ONH biomechanics.^11^^–^^13^ Therefore, assessment of scleral biomechanical properties is essential for understanding the progression of several ocular diseases.^14^^,^^15^

Sclera can be categorized as a highly hydrated tissue like cornea.^16^^–^^18^ The water content of porcine sclera is about 200%, and that of human sclera is about 250%.^19^ The main structural component of sclera is collagen, but it also contains charged proteoglycans (PGs) and mobile ions. Glycosaminoglycans (GAGs) are highly negatively charged chains attached to PGs, and they are expected to contribute to the structural behavior of the sclera. GAGs primarily regulate volumetric behavior through their prominent role in generating swelling pressure inside the ECM (Fig. 1).^17^ Swelling pressure is associated with scleral osmotic pressure, which results from the interaction of mobile ions with GAGs that are distributed throughout the tissue in essentially fixed positions. The greater the concentration of GAGs in hydrated soft tissues, the greater the concentration of ions and the greater the resultant osmotic pressure will be.^20^^,^^21^ GAG content begins decreasing in the sclera with increasing age; furthermore, it undergoes alterations due to ocular diseases such as glaucoma.^22^ A decrease in GAG-digesting enzymes has also been noted in conditions such as nonophthalmic and highly hypermetropic eyes.^3^ Furthermore, several studies have shown that the scleral GAG content changes during normal growth, and it may contribute to the regional alterations in the scleral biomechanical properties.^23^^,^^24^

**Figure 1.**
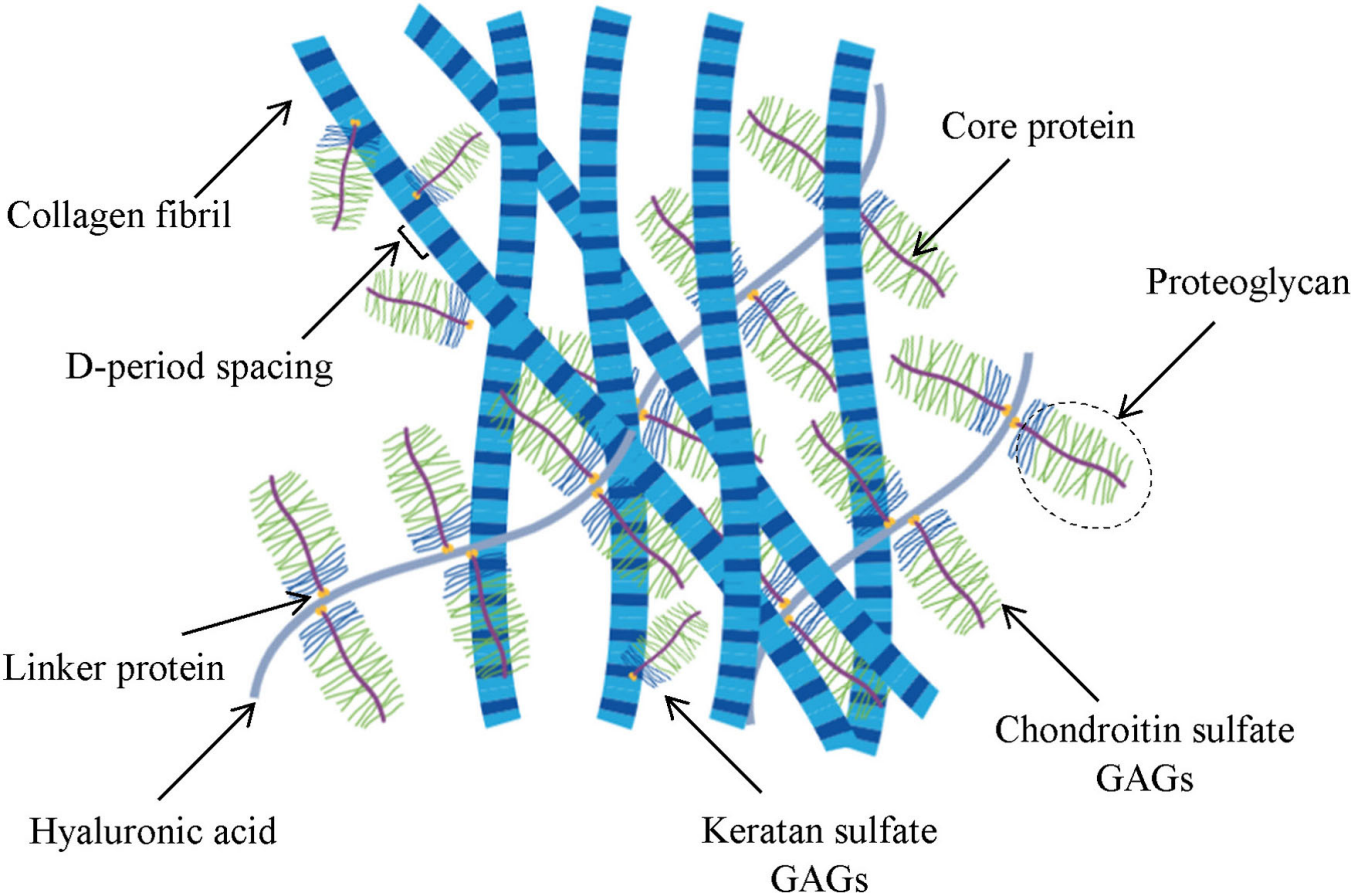
The interaction between GAG and collagen in the ECM is thought to play an important role in the function and mechanical properties of soft tissue. In addition to collagen fibers, the main load-bearing component of the ECM, it is thought that GAGs also play a mechanical role.

The unique structure and arrangement of the ECM provide the sclera with the considerable viscoelastic properties necessary to fulfill its required functions. Moreover, the regional properties of sclera originate from its specific ECM; for example, the swelling ratio of the anterior section of the sclera was documented to be lower than that of the posterior sclera.^25^ Along with regional hydration differences, human sclera has a non-uniform thickness. The scleral thickness is the thickest at the posterior pole, gradually decreasing to its thinnest values adjacent to the equator and expanding again toward the limbus.^26^ The thickness changes have been attributed to the microstructure of the scleral ECM.^27^ Previous studies have investigated the equilibrium hydration–GAG relationship in human,^28^ rabbit,^29^ and porcine^30^ sclera, but regional differences were not examined.^25^ In a recent study, we characterized the effects of GAGs on the tensile properties of posterior porcine sclera without directly measuring their hydration.^31^ Here, we investigated the effects of GAGs on scleral swelling and biomechanical properties by meticulously tracking the changes in hydration and thickness over time, as well as by performing uniaxial tensile stress relaxation tests. Furthermore, we characterized the possible regional differences in scleral properties by using samples from posterior and anterior regions.

## Materials and Methods

### Sample Preparation

**Figure 2.**
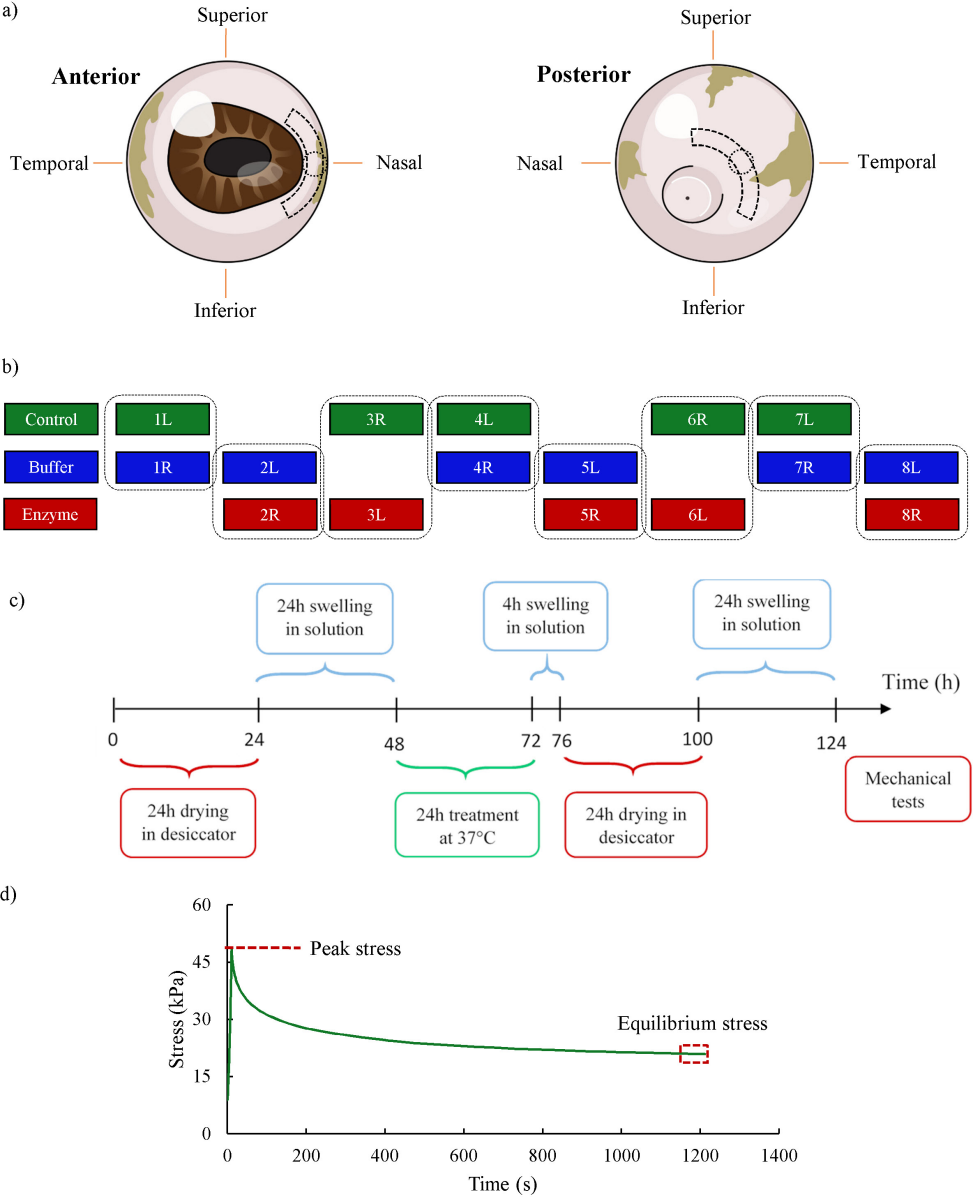
(**a**) Anterior and posterior strips were dissected from the eyeballs in the superior–inferior direction. The discs, intersecting with the center of the strips, were cut for GAG content measurement studies. (**b**) The assignment of the strips from eyeballs to the different groups. The label of each strip shows the number of the eyeball pairs and whether it was from the left (L) or right (R). (**c**) The diagram shows the experimental protocol. All samples were initially dried for 24 hours. They were then immersed in a PBS or buffer solution, depending on the specific group to which they belong, to swell again before undergoing the required treatment process for 24 hours at 37°C. After treatment, the samples in the buffer and enzyme group were immersed in a buffer solution and the samples in the control group were immersed in PBS for 4 hours before being dried for 24 hours. The samples were then allowed to swell for 24 hours before their mechanical properties were measured. (**d**) The stress–relaxation response of a typical scleral strip. The peak stress occurred at 5% strain; the equilibrium stress was defined as the average stress over the last minute of the relaxation period.

Pairs of eyes were obtained from a local slaughterhouse within 4 to 6 hours after pigs were slaughtered. Two strips (each, 4 × 12 mm^2^) were immediately dissected from the anterior and posterior regions and in the superior–inferior direction (Fig. 2a). The posterior strips were along the posterior pole, and the anterior ones were adjacent to the nasal side of the limbus. Additionally, 5-mm scleral discs were punched from separate eyeballs in order to measure the GAG content. These discs were obtained from eyeballs such that their center coincided with the center of anterior and posterior strips (Fig. 2a).

### Experiments

#### Swelling Procedure

Eight pairs of porcine eyes were assigned to three groups of study: (1) PBS treatment (control), (2) buffer treatment (buffer), and (3) enzyme treatment (enzyme). The extracted strips were divided into these three groups to provide control–buffer and buffer–enzyme comparisons, as shown in Figure 2b.

The hydration was defined as the water weight divided by the dry weight. In order to determine the initial hydration of the samples, the wet weight was first measured by an analytical balance. The samples were then dried for 24 hours in a desiccator; a preliminary study showed that 24 hours is long enough to obtain the dry weight of the samples. After measurement of the dry weight of each specimen, they were placed in a solution for 24 hours to rehydrate at room temperature. The solution used for control samples was PBS, and samples from the buffer and enzyme groups were immersed in the Trizma buffer solution (Sigma-Aldrich, St. Louis, MO, USA). During this period, the wet weight of each specimen was measured after 1, 10, 20, and 30 minutes followed by measurements at 1, 2, 3, 4, 12, 18, and 24 hours. Then, the samples in the control, buffer, and enzyme groups were put in a PBS, buffer, or chondroitinase ABC (ChABC) enzyme solution, respectively, at 37°C for 24 hours. The wet weight of samples in all groups was measured after the samples were washed five times and again after they were placed in the solution for another 4 hours. The washing step was necessary to remove GAGs from the samples and was done for samples in all other groups in order to create similar conditions. The samples were then dried again for 24 hours and swelled in PBS (control group) or in Trizma buffer (buffer and enzyme treatment groups) for 24 hours. This dehydration and rehydration step was included to assess the possible effect of GAG removal on the swelling response of the samples. Four thickness measurements were taken by a digital pachymeter (1) after dissection, (2) after the first 24-hour swelling, (3) after 24-hour treatment followed by 4-hour swelling, and (4) after the second 24-hour swelling.

#### Degradation Method and GAG Content Assay

Eight sclera discs excised from the anterior and posterior regions (Fig. 2a) were divided into buffer and enzyme treatment groups. Following the protocol described in our recent study and similar ones,^30^^–^^32^ the enzyme group of specimens was treated enzymatically in 0.125 U/mL ChABC to remove all of the GAGs. The scleral strips were incubated under gentle agitation for 24 hours at 37°C in a buffer solution containing 50-mM Trizma base (pH 8.0) with 60-mM sodium acetate and 0.02% BSA. Excess GAGs were detached from scleral tissue by washing the strips in the buffer immediately after the treatment. Specimens in the buffer group were immersed in Trizma buffer alone for 24 hours. This procedure was similar to what was done to remove GAGs from the scleral strips. The reduction in GAG content in the enzyme group was compared to that of the buffer-treated discs. The GAG content (µg/mg dry weight) was determined by biochemical assay (Biocolor Ltd., Carrickfergus, UK).^31^

#### Viscoelasticity Measurement

After the hydration study, a uniaxial stress relaxation tensile test was conducted with a RSA-G2 solids analyzer (TA Instruments, New Castle, DE). The specimens were mounted into the grips of the analyzer using sandpaper; no slippage or failure at the grip interface was observed. PBS, as the bathing solution, ensured that the scleral strips remained hydrated for the duration of tensile tests; because the samples reached equilibrium hydration prior to the mechanical test, no hydration effect was expected on the mechanical measurements.^9^^,^^18^^,^^19^^,^^33^^,^^34^ A 0.01-MPa tare stress was applied to remove the slack in the strips and to define the reference length or the 0% strain point. The strips were then subjected to the stress relaxation experiment with an engineering strain of 5%, a steady displacement rate of 2 mm/min, and a 20-minute relaxation time. Based on the recorded load and reference configuration, the equilibrium stress was calculated as the average of stress over the last minute of the relaxation period (Fig. 2d). Furthermore, the peak stress was the tensile stress at 5% strain, and the engineering strain was determined from dividing the elongation during the uniaxial tensile test by the reference length. No preconditioning was included in the mechanical testing protocol.^35^

### Statistical Analysis

The *t*-test and ANOVA test were used to verify the statistical significance of the comparisons of the hydration, thickness, biochemical, and biomechanical parameters among the various experimental groups. All comparisons with *P* ≤ 0.05 were considered significant. All values are reported as the mean ± standard deviation.

## Results

### GAG Content, Swelling, and Thickness

#### GAG Content

The GAG content of the anterior and posterior scleral samples was obtained for the buffer and enzyme groups (Table 1). Regional GAG content was significantly different (*P* < 0.0001), as the posterior specimens had, on average, 101.5% higher GAG content than the anterior samples. The GAG content was significantly different between the buffer and enzyme groups in both anterior and posterior samples. Enzyme treatment decreased the GAG content, on average, by 96.9% (*P* < 0.001) and 89.3% (*P* < 0.0001) in the anterior and posterior discs, respectively, compared to the buffer group.

**Table 1.** Comparison of GAG Content of Buffer-Treated and Enzyme-Treated Specimens

	Group (µg/mg Dry Tissue Weight), Mean ± SD	
	Buffer-Treated	Enzyme-Treated
Anterior	3.21 ± 0.34	0.1 ± 0.02
Posterior	6.46 ± 0.24	0.69 ± 0.2

#### Swelling

**Figure 3.**
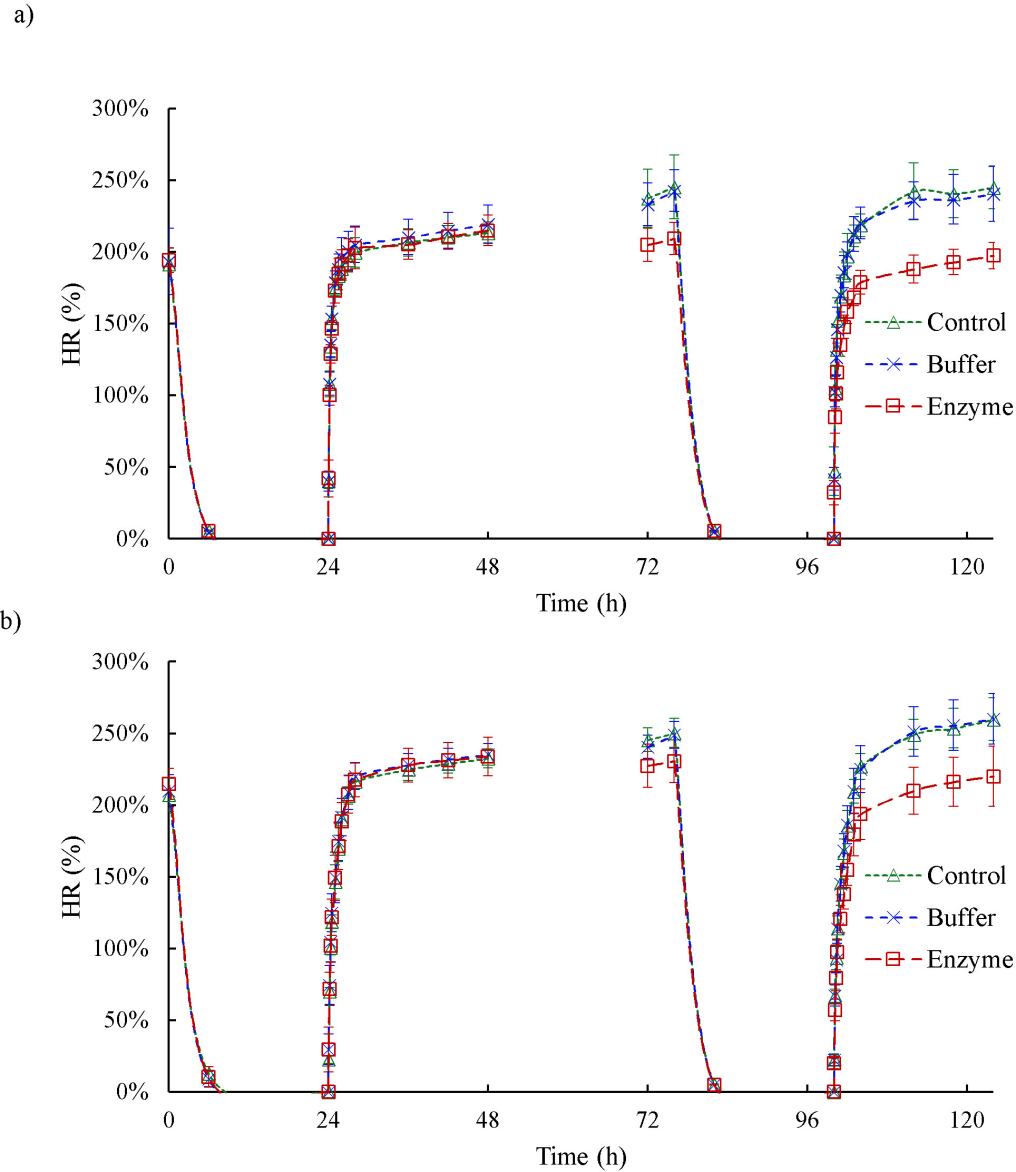
The results of the hydration study on (**a**) anterior strips and (**b**) posterior strips; the plot shows the water content (%) of samples over the duration of the experiment. The *symbols* and *vertical bars* represent the mean and standard deviation, respectively.

**Figure 4.**
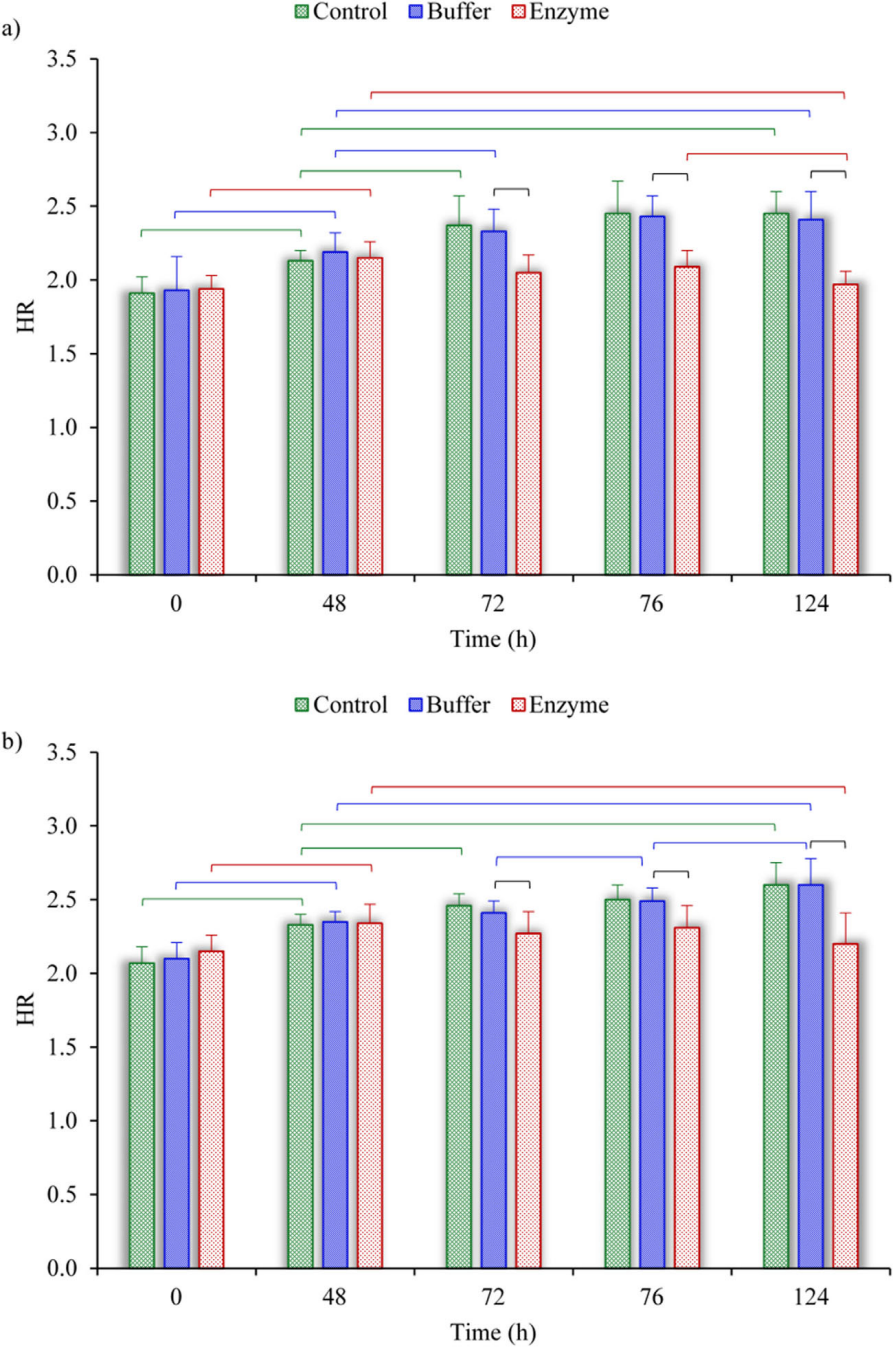
Comparison of the hydration ratios among the control, buffer, and enzyme groups of (**a**) anterior strips and (**b**) posterior strips. The horizontal brackets indicate significant differences (*P* < 0.05) among the different groups. The data are shown for the critical times of 0, 48, 72, 76, and 124 hours, corresponding to immediately after dissection, after the first 24-hour hydration, after 24-hour treatment, after 4-hour hydration, and after the second 24-hour hydration, respectively (see Fig. 2c).

 Figure 3 depicts the results of the hydration tests conducted on anterior and posterior strips by displaying the water content of samples in percentage over time. The scleral hydration was obtained for the control–buffer and buffer–enzyme comparisons in the anterior and posterior sclera (Fig. 4). After dissection, average hydration was 8.1% higher in the posterior samples than in the anterior samples (*P* = 0.02); after the first 24 hours of hydration, the hydration of the posterior strips was 9.1% greater than for the anterior strips (*P* < 0.001). No significant difference was found between point-to-point hydrations in control–buffer comparisons. Hydration was the same for the buffer and enzyme groups before treatments. In the anterior specimens, enzyme treatment decreased hydration immediately after the treatment by 12.2%; after 4 hours of swelling by 13.8%; and after the second rehydration by 18.0%. For the samples obtained from the posterior region, the reductions were 5.5%, 7.4%, and 15.5%, respectively.

#### Thickness

**Figure 5.**
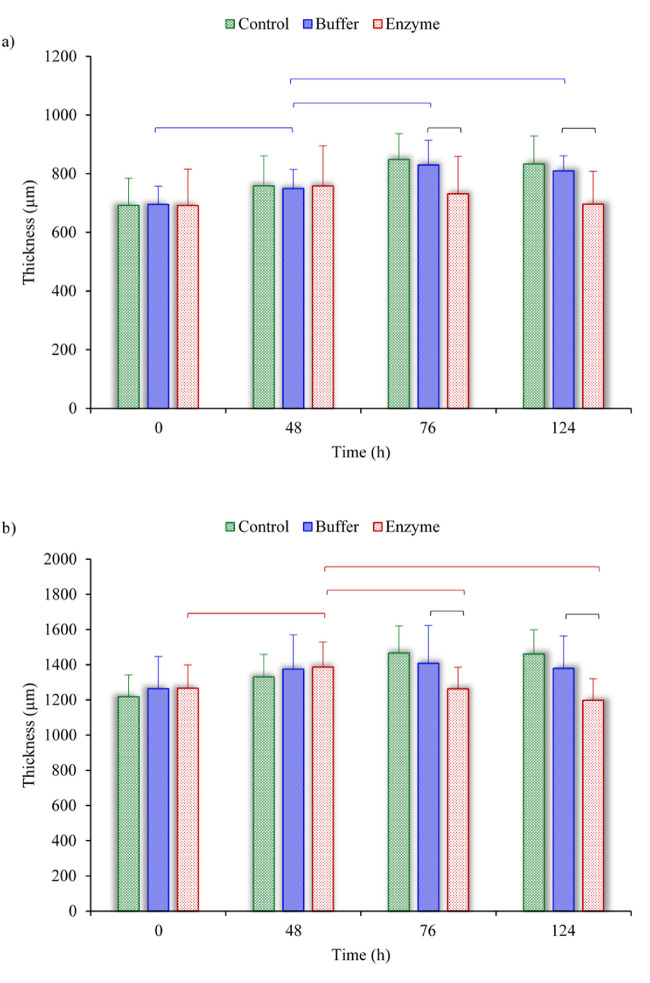
Comparison of thickness variation among the control, buffer, and enzyme groups of (**a**) anterior strips and (**b**) posterior strips. The horizontal brackets indicate significant differences (*P* < 0.05) among the different groups. The data are shown for the critical times of 0, 48, 76, and 124 hours, corresponding to immediately after dissection, after the first 24-hour hydration, after 4-hour hydration, and after the second 24-hour hydration, respectively (see Fig. 2c).

The average thickness was measured for samples in the control, buffer, and enzyme groups at four stages. Figure 5 shows the thickness variation for the anterior and posterior strips. In all comparisons, no significant difference was observed between the thickness of samples in the control and buffer groups. The same was true for the thickness of samples in the buffer and enzyme groups except at 76 and 124 hours. After the second rehydration, the thickness was 14.0% (anterior) and 13.1% (posterior) smaller in enzyme treatment than in buffer treatment groups (*P* < 0.05).

### Mechanical Behavior

We compared the peak and equilibrium stress from the stress relaxation experiments between control and buffer groups (Table 2). There was no significant change in control–buffer comparisons of anterior and posterior strips. Compared to the buffer group, the enzyme-treated specimens showed a significantly larger peak and equilibrium stress for anterior and posterior (Table 2).

**Table 2.** Comparison of Peak and Equilibrium Stress Between the Control and Buffer Groups and Between the Buffer and Enzyme Groups

	Anterior (kPa), Mean ± SD					Posterior (kPa), Mean ± SD				
				*P*					*P*	
	I Control Group	II Buffer-Treated Group	III Enzyme-Treated Group	I, II	II, III	IV Control Group	V Buffer-Treated Group	VI Enzyme-Treated Group	IV, V	V, VI
Peak stress	43.3 ± 20.4	54.2 ± 28.5	143.5 ± 51.6	0.25	<0.005	67.5 ± 29.3	85.3 ± 13.5	121.1 ± 13	0.13	<0.005
Equilibrium stress	6.3 ± 1.2	7.2 ± 3.2	21.6 ± 9.4	0.27	<0.05	16.7 ± 9.6	28 ± 8.4	45 ± 7.7	0.08	<0.005

## Discussion

GAGs play a role in the growth and maintenance of sclera. These large water‐binding molecules occupy the space between the collagen fibrils and are assumed to be involved in regulation of the fusion and diameter of neighboring fibrils.^31^ Scleral swelling capability is accordingly supposed to be closely linked to the composition of the ECM.^36^ Moreover, it has also been reported that GAGs may be related to glaucoma and myopia.^32^ In the present study, the GAG content, hydration, thickness, and tensile properties of scleral samples obtained from anterior and posterior parts were characterized to explore how GAGs contribute to the microstructural and biomechanical integrity of sclera. By utilizing a comprehensive protocol, we obtained results that could extend our knowledge of the contribution of GAGs to the structural and tensile properties of scleral tissue.

The findings of the present study (Table 1) are in line with previous studies on human sclera,^37^^,^^38^ which have indicated that the relatively thinner sclera in anterior has a low GAG content.^39^ In contrast, thicker tissue and higher GAG content were seen in the peripapillary or posterior sclera. Furthermore, chondroitin sulfates were reported to be most abundant in the sclera near the posterior pole. In our study, the posterior porcine specimens had a higher content of GAGs (6.46 ± 0.24 µg/mg) compared to the anterior specimens (3.21 ± 0.34 µg/mg). These levels of GAG contents are in general agreement with previous studies that have reported the same difference between anterior and posterior regions of human sclera.^25^^,^^37^ Our preliminary digestion study showed that 0.125 U/mL of enzyme concentration and 24 hours of treatment are sufficient to degrade primary GAGs, such as dermatan sulfates and chondroitin sulfates, from both anterior and posterior sections. The treatment time of 24 hours was determined based on GAG content quantification indicating more than 90% degradation. Successful digestion was reached for the posterior section even after 18 hours^31^; we extended this incubation time by 6 hours in order to ensure that GAGs were digested from anterior samples, as well. We note here that our preliminary studies suggested that a higher concentration of ChABC might not significantly influence the required incubation time. Furthermore, the treatment with ChABC did not completely remove all of the GAGs (Table 1). This is because ChABC primarily digests chondroitin-4 sulfate, chondroitin-6 sulfate, and dermatan sulfates, but other GAGs, such as keratan sulfate, also exist in the sclera.^38^ Furthermore, incomplete enzyme diffusion and penetration throughout the samples may have left some chondroitin and dermatan sulfates in the digested tissues. The low density of the remaining GAGs in the digested strips (Table 1) is not expected to make an experimentally measurable difference, but future studies are required to fully determine the effects of the residual GAGs.^40^

The hydration of the sclera is expected to vary based primarily on its GAG density.^41^ The negative charges of GAG side chains, because of their hydrophilic properties, regulate diffusional transport and affect the intrascleral swelling pressure.^42^ Boubriak et al.^25^ showed that the posterior human sclera is capable of swelling more than the anterior section, possibly because of differences in the GAG density of these two regions. We also observed that the average hydration of posterior samples was greater than that of the anterior samples; however, this difference was not always significant. Furthermore, we also observed that the hydration level was independent of the bathing solution (PBS or buffer) for all specimens. These two bathing solutions have comparable ionic strengths, which could explain why they caused similar swelling effects in the sclera.^43^ All specimens in the enzyme group underwent 4 hours of incubation in a non-enzymatic solution in order to assess their hydration following GAG removal. The hydration levels of both regions were significantly lower than that of the controls, because GAGs were removed from the enzyme group. Note that scleral hydration is expected to be a function of GAG density.^41^^,^^44^ Specifically, Brown et al.^41^ demonstrated a direct relationship between human scleral hydration and sulfated glycosaminoglycans. In this study, we included a second dry-swelling sequence in order to further confirm the effect of GAGs on hydration (i.e., a significant difference in hydration between the buffer and enzyme groups). However, an insignificant change in hydration after ChABC digestion was reported in rabbit sclera.^29^ Furthermore, Boubriak et al.^25^ reported an insignificant decrease in the hydration of human sclera because of GAG removal. Murienne et al.^32^ also reported a decrease in the hydration of human sclera because of GAG depletion, but only for posterior samples from certain regions. An increase in the hydration of porcine samples was also seen in a previous study and was explained in terms of the volume that GAGs and water molecules occupy,^30^ a hypothesis that has yet to be investigated. We believe that the reason for the inconsistency among previous reports is because of differences in species, differences in experimental methods, and the low concentration of scleral GAGs. We note, however, that the findings of the present study agree with the expectation that GAG-depleted scleral samples should swell less than their controls because they have fewer GAGs.

The swelling ability of porcine specimens was measured before and after incubation in three different solutions so we could fully investigate the possible effects of GAGs. After the samples were incubated in solutions without the ChABC enzyme, an insignificant difference was observed between pre- and post-treatment equilibrium hydration (Fig. 3). The initial hydration of all specimens (at 0 hour) was significantly smaller than their hydration after the first drying and rehydrating cycle (at 48 hours). The 24-hour treatment of samples in the control and buffer groups significantly increased their hydration, except for the posterior samples of the buffer group, where only an increase in hydration was observed. This suggests that the longer the tissue is immersed in the solution, the more it swells; that is, free bound water molecules enter the ECM and increase collagen fiber spacing.^30^ The 24-hour enzyme treatment decreased the average hydration of the samples, as removing GAGs from the samples decreased their swelling tendency.

In order to better investigate the effect of GAG removal on swelling properties of the samples, the strips were dried and rehydrated again for 24 hours. Comparing the hydration of samples with their hydration after the first drying and hydrating step, we observed that hydration in the enzyme group decreased significantly, but hydration of the other samples increased. The significant increase in the hydration of samples in the buffer and control groups could be explained as follows. The sclera ECM has a relatively high collagen content and disorganized large intertwined collagen fibrils that form bundles.^43^ The packing of collagen fibrils possibly plays a role in the ability of the sclera to swell. The multiple dehydration and rehydration of the samples may have affected their ECM microstructure and reduced their ability to resist against expansion of the interfibrillar space caused by the water that is drawn to the tissue by GAGs. This argument agrees with previous studies suggesting a correlation between interfibrillar spacing and scleral hydration.^43^ In this regard, the variation of rehydration capacity of the tissue after dehydration should also be noted.^45^ Here, we subjected the samples in all groups to the same experimental protocol and observed a significant reduction in the hydration of samples in the enzyme group, primarily because the majority of GAGs had been removed due to the enzyme treatment process, and the swelling tendency was proportional to the density of GAGs inside the tissue. A few artifacts were reported for long-term incubation in bathing solution (PBS and Tris-based buffer), including chemical effects linked to the diffusion of solutes into the tissue.^46^ Nonetheless, the trends were similar for samples in the control and buffer groups, suggesting that those effects should not affect the main findings of the present work.

We also tracked the thickness of each specimen at four different times: (1) after dissection, (2) after the first 24-hour hydration, (3) after 24-hour treatment followed by 4-hour hydration, and (4) after the second 24-hour hydration. The significant decline in the mean thickness comparing the ChABC-treated group to the buffer-treated group suggests a possible collagen fibril rearrangement. The decrease in thickness might also be influenced by the increase of collagen fiber fusion. This can be because the loss of GAGs creates greater free intrafibrillar space and less extrafibrillar space by decreasing the degree of steric exclusion.^25^^,^^32^ Weak repulsive electrochemical forces after GAG removal may result in the collagen fibrils being stacked up on top of each other and a reduction in the lateral fusion of adjacent fibrils. Collagen fibrils in the decorin-degraded skin tissue have demonstrated a similar merging, which was observed by scanning electron microscopy and atomic force microscopy^47^; however, such studies were not done in the present study. Furthermore, scleral thinning is observed in various conditions such as high myopia^13^^,^^48^^,^^49^ and glaucoma.^16^^,^^50^^,^^51^ Also, with increasing the age, the sclera becomes thinner, although the collagen fibers become thicker and less uniform.^2^

The stress relaxation test results showed less relaxation for enzyme specimens in both anterior and posterior regions. Both peak and equilibrium stress showed a difference in relaxation behavior. The stiffer behavior can be directly associated with lower swelling in GAG-depleted specimens compared to either the control or buffer-treated strips. Collectively, these results imply that GAGs interact with the collagen fiber bundles to affect scleral viscoelasticity. One explanation for this observation is that the lack of pre-stress in the collagen fibers of GAG-digested sclera may account for an increase in the stiffness, similar to what has been suggested for ChABC-treated cartilage.^52^ The close agreement of the findings of the present study with the literature on connective tissue^53^^–^^55^ suggests that GAG–collagen interactions regulate the relaxation response of sclera by fiber recruitment. The increased stiffness of scleral tissue due to the ChABC treatment was more pronounced in posterior sclera than in anterior sclera. The difference in mechanical properties between enzyme-treated anterior and posterior sclera may be related to the architecture of fiber organization. The findings are compatible with a previous study,^25^ which reported that changes of scleral GAGs affected the anterior region less than the posterior part. Also, the size of the interfibrillar spacing is different in these two regions.^3^ The outcomes of various studies indicate that GAG content, fibrillar organization, and orientation are crucial for the different behaviors of anterior and posterior sclera.^25^^,^^56^ For example, the posterior human sclera was shown to have wide, loosely woven collagen fiber bundles, in contrast to the thinner, more tightly interwoven fiber bundles in the anterior region.^56^

One limitation of the current hydration study is that the samples were swelled at room temperature (20°C–22°C), which differs from internal body temperature^57^; however, the same conclusion can be drawn for what occurs at body temperature.^58^ Any possible temperature effects would be expected to be consistent for all scleral groups; thus, this limitation should not have affected the main conclusions. Another drawback, however, is the mechanical tests used to measure the mechanical properties of the scleral strips. The uniaxial tensile tests do not represent the natural loading conditions of the scleral samples. To measure the regional hydration differences, we had no alternative but to dissect a section of the sclera; therefore, scleral strips were cut to run the uniaxial test at the end of hydration study. Although the advantages and disadvantages of uniaxial to inflation experiments have been discussed in previous studies,^9^^,^^27^^,^^31^ we emphasize here that uniaxial tensile tests determine the mechanical response of sclera in a nonphysiological loading condition. Thus, its measurement may not be an accurate representation of the mechanical properties of the sclera in vivo. The tensile tests are best suited for conducting comparative studies focusing on investigating the effect of different parameters. Moreover, the results of the present study may not be used for human samples, because the effects of GAG digestion could be different for porcine and human sclera.^30^^,^^32^ Repetitive dehydration and swelling tests of the sclera may have induced small damages that influenced the measured viscoelastic changes to the specimens. However, we tested a few samples that were equilibrated in PBS for 5 hours using the same mechanical protocol and found no significant difference (Supplementary Fig. S1); thus, it can be concluded that the postmortem testing may not have significantly affected the tensile stress response of the samples. Furthermore, the stress at 5% strain obtained from this study for posterior sclera was in an overall agreement with stress levels that were reported previously for porcine samples.^28^^,^^31^^,^^35^^,^^59^^,^^60^ A direct comparison cannot be made because of differences in sample preparation and testing protocols between different studies. Finally, this study was intended to investigate hydration-related changes caused by GAG depletion and was not designed to replicate in vivo conditions. Thus, it may be problematic if measurements from the present study, as well as those from other studies that have used the uniaxial testing method, are directly used as in vivo mechanical properties of scleral tissue; material parameters found from the inflation or biaxial testing methods might be more appropriate, as these tests provide a better representation of the scleral natural loading condition.

In conclusion, we studied the contribution of GAG removal to hydration, thickness, and viscoelastic properties of porcine sclera in anterior and posterior regions. The main finding of this study is that GAGs are responsible for the swelling process and drawing water into the tissue. We observed that microstructural changes in scleral ECM alter the properties of the sclera. Although a few studies in the past have reported contradictory results for GAG depletion in the sclera, this study is novel in that it combines measuring hydration and mechanics to identify the effects of GAGs on scleral mechanical and swelling properties. We also found that the swelling properties of sclera differ regionally. Our results might be useful for future studies regarding drug delivery into the vitreous humor through sclera, where the hydration and mechanical properties of the sclera are important. Also, the findings here may help researchers better explain the effects of ocular disease on the properties of the sclera, such as why glaucomatous eyes have thinner and stiffer sclera than normal eyes.

## Supplementary Material

Supplement 1
