# Supplementary material for: Regional Differences in the Glycosaminoglycan Role in Porcine Scleral Hydration and Mechanical Behavior
Source: Invest Ophthalmol Vis Sci. 2021 Mar 22;62(3):28. doi: 10.1167/iovs.62.3.28 (PMC7991977; doi:10.1167/iovs.62.3.28)
Supplement: Supplement 1 [file iovs-62-3-28_s001.pdf]

The specimens in the present study went through a number of dehydration in the desiccator and rehydration in PBS before being mechanically tested. In order to determine the possible effect of this long-term treatment process on the mechanical properties of the samples, we tested five posterior samples that were equilibrated in PBS only for 5 hours after dissection using the exact same testing protocol. Figure S1 compares the stress-strain and stress-relaxation response of these samples with those found for the samples in the control group. No significant difference was observed between the maximum stress and equilibrium stress ($P > 0.15$), which suggests that the long treatment period should not have had any significant effect on the mechanical response of the specimens.

a)

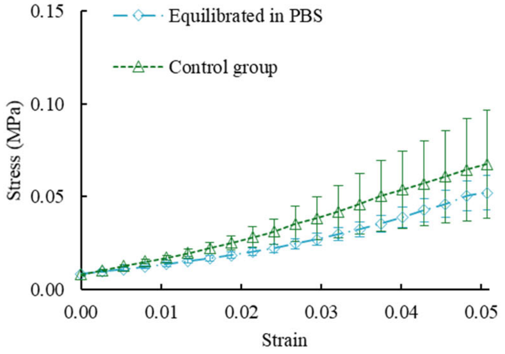

b)

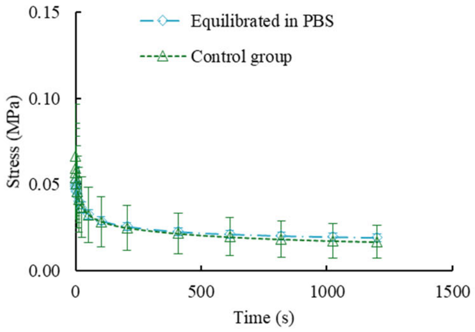

Figure S1. *a) Stress-strain and b) stress-time curves comparing the response of posterior samples from the control group with the strips tested after being equilibrated in PBS for only 5 hours after dissection.*
